# Measuring implementation climate: psychometric properties of the Implementation Climate Scale (ICS) in Norwegian mental health care services

**DOI:** 10.1186/s12913-021-07441-w

**Published:** 2022-01-04

**Authors:** Nadina Peters, Randi Hovden Borge, Ane- Marthe Solheim Skar, Karina M. Egeland

**Affiliations:** Norwegian Centre for Violence and Traumatic Stress Studies (NKVTS), Gullhaugveien 1, 0484 Oslo, Norway

**Keywords:** Implementation climate, Evidence-based practice, Mental health services

## Abstract

**Background**

Employees’ perceptions of organizational climate for implementation of new methods are important in assessing and planning for implementation efforts. More specifically, feedback from employees’ points to which implementation strategies to select, adopt, and tailor in building positive climate for implementation of new evidence-based practices within the organization. Implementation climate can be measured with the Implementation Climate Scale (ICS). The purpose of this study was to investigate the psychometric properties of the Norwegian version of the ICS in outpatient mental health clinics.

**Methods**

The ICS was administered to 383 clinicians within 47 different child and adult mental health clinics across the country. We conducted confirmatory factor analysis to assess the psychometric functioning of the ICS. Cronbach’s alpha was examined to assess internal consistency. We also examined criterion related validity of the scale by comparing it with an alternative measure of implementation climate (concurrent validity) and by examining correlations with clinicians’ intentions to use evidence-based practices.

**Results**

Results supported the 6-factor structure and the internal consistency reliability of the ICS. One exception was poor functioning of the Reward scale. Concurrent validity was stronger at the group than at the individual level, and assessment of associations with clinicians’ intentions to use evidence- based practices showed positive correlations.

**Conclusions**

The Norwegian version of the ICS is a promising tool for assessing implementation climate which can provide organizations with specific feedback concerning which aspects of the implementation climate to attend to. Due to poor functioning of the Reward scale, adaptations and further testing of this is recommended.

**Supplementary Information:**

The online version contains supplementary material available at 10.1186/s12913-021-07441-w.

## Background

Creating a positive climate for implementation of evidence-based practices (EBPs) is critical for these practices to be implemented successfully [1] and ultimately improve quality of services for patients in mental health care settings. Strategic implementation climate, as originally defined by Klein and Sorra [2], refers to “targeted employees’ shared summary perceptions of the extent to which their use of a specific innovation is rewarded, supported, and expected within their organization”. Furthermore, employees’ perceptions “are the result of employees' shared experiences and observations of, and their information and discussions about, their organization's implementation policies and practices” (2, p. 1060). Implementation climate is associated with implementation effectiveness [3–7].

Measuring employees’ perceptions of how organizations value EBP implementation can give important insight into factors that need to be attended to foster a good implementation climate within the organization. Building on the original definition of implementation climate [8], Ehrhart, Aarons [9] sought to further break down climate dimensions. They defined EBP implementation climate as “employees’ shared perceptions of the importance of EBP implementation within the organization”. As research shows that leaders play an important role in creating a strategic implementation climate [4, 10, 11]. They also sought to capture how employees perceive what leaders communicate as valued within the organization through their actions, policies, practices and processes [12]. Ehrhart, Aarons [9] developed the Implementation Climate Scale (ICS) to better understand and make use of feedback about specific behaviors and strategies within an organization, setting the stage for implementation efforts in mental health services. The ICS covers six dimensions: 1) how teams/organizations focus on EBPs, 2) whether organizations provide educational support for EBPs, 3) if employees receive recognition for using EBPs, 4) are the employees rewarded for using EBPs, 5) is the staff selected based on experience with EBPs, and 6) is the selection of staff based on their general openness to adopt new EBPs.

The ICS has demonstrated good psychometric properties in several settings [9, 13–16]. Reliability testing has found overall good internal consistency in mental healthcare settings in the USA, with a Cronbach’s alpha of 0.91, and subscales values ranging from 0.81- 0.91 (focus on EBP: 0.91, educational support for EBP: 0.84, recognition for EBP: 0.88, rewards for EBP: 0.81, selection for EBP: 0.89, and selection for openness: 0.91). Although the six-factor structure of the ICS has been confirmed, the Reward subscale showed lower correlations with the other five dimensions of the scale at the individual level in substance abuse settings (*average r* = 0.25) [15] and child welfare settings (*average r* = 0.23) [14] compared to mental healthcare setting in the USA (*average r* = 0.32) where it was originally developed [9]. In an educational setting in the USA [13] the Reward subscale was removed from the final ICS model to improve the model fit.

Ehrhart, Aarons [9] found that the ICS correlated with other measures of relevant constructs within organizational climate such as service climate (strategic climate), organizational climate (molar climate) and organizational readiness for change, but that it also was distinct in measuring *implementation* climate. Another measure of implementation climate was developed about the same time as the ICS, Implementation Climate Measure (ICM) [3]. The ICM captures employees’ perceptions of overall implementation climate across three climate dimensions (rewarded, supported and expected). As Ehrhart, Aarons [9] point out, there are differences and similarities in these two measures of the same construct, most notably ICS being more specific to factors related to policies, practices, and procedures within the organization. The authors argue for practical use of the ICS in implementation processes in providing feedback to organization on employees’ perceptions related to these specific factors.

The ICS has also shown positive correlations with implementation outcomes, such as knowledge and attitudes toward EBPs [11, 17], intensity of supervisory focus on evidence-based treatment [6], and clinicians’ use of EBPs [11]. As measuring actual use of EBPs is challenging [18], measuring clinicians’ intention to use newly learned EBP can be a good alternative, as intentions are proposed to be determinants of behavior [19–21] and thus important when making a decision to adopt EBPs.

This is the first study to investigate the psychometric properties of the ICS in a mental health care setting outside of the USA. First, we will examine whether the six-factor structure of the ICS can be confirmed in a sample of clinicians within outpatient mental health clinics. Secondly, we will examine the internal consistency of the total scale and subscales. Lastly, we will assess the criterion-based validity of the scale by examining its associations with an alternative measure of implementation climate, namely the Implementation Climate Measure (ICM) [3], and clinicians’ intentions to use EBPs through the Measure of Innovation-Specific Implementation Intentions (MISII) [22].

We hypothesize that the use of the Norwegian version of the ICS within a mental health context will show:

1. The same six-factor structure as the original version (i.e., measurement model validity)
2. Internal consistency as a whole and for all subscales (i.e., reliability)
3. A positive correlation with the alternative measure of implementation climate (i.e., concurrent validity)
4. A positive correlation with clinicians’ intentions to use EBPs (i.e., criterion-related validity)

## Method

### Setting

This study is part of an ongoing national implementation of evidence-based treatment for post-traumatic stress disorder (PTSD) in Norwegian outpatient mental health clinics for children and adults [23]. The study was approved by the Norwegian Centre for Research Data (ref. nr. 60,059/3/OOS and 60,036 / 3 / LH) and it was retrospectively registered 25.10.2018 in ClinicalTrials with ID: NCT03719651.

The Norwegian health care system is semi- decentralized and consists of specialist and community care. The specialist health care system is a responsibility of the Norwegian Ministry of Health and Care Services that is administered through the state enterprises called Regional Health Authorities. The actual care is performed by subsidiary regional health trusts, and all treatment are without costs for the patients There are 43 health trusts in Norway each responsible for one or more hospitals that include both inpatient and outpatient specialized mental health care services. Child and adolescent clinics (BUP) are outpatient clinics for children up to age 18 and their families. There are 87 BUPs in Norway. District psychiatric centers (DPS) consist of both outpatient and inpatient services for adults above the age of 18. There are 75 DPSs in Norway. In 2019, 4.6% of the population received specialized mental health services [24].-Child and adolescent outpatient clinics are implementing Trauma-Focused Cognitive Behavioral Therapy (TF- CBT) [25] and the adult outpatient clinics are implementing Eye Movement Desensitization and Reprocessing therapy (EMDR) [26] and Cognitive Therapy for Post-Traumatic Stress Disorder (CT- PTSD) [27].

### Procedures

Regional health trusts recieved an e-mail with information about the project and invitation for mental health outpatient clinics to participate. Those clinics that responded with interest to participate were then contacted directly by the research team. Leaders at each clinic received further information about participation through e-mail, telephone contact, and a face-to-face meeting with the research team. All clinics that made the decision to participate were included in the project.

After the inclusion and contract signing, the clinic leaders informed their staff about participation in the project. The research team held a meeting with all clinicians and their leaders at each clinic. The goal of this meeting was to inform all the staff about the project and train all clinicians in screening of trauma exposure and posttraumatic stress symptoms. The leaders of the clinics were responsible to provide the clinicians that were not present at this meeting with the link to a training video and the screening material. Leaders were further instructed to select clinicians for training in the EBPs for PTSD. All clinicians at the clinics were invited to participate in the survey. Administrative staff were not included in the survey. The Leadership and Organizational Change for Implementation (LOCI) [10] model was used as an implementation strategy [23]. Clinics that had leaders who did not agree to participate in LOCI were not eligible to participate in the project. All leaders agreed to participate in LOCI.

The ICS was one of several scales that were administered through online surveys sent to the respondents via email. An email reminder was sent to participants who had not yet responded 14 and 28 days after the first invitation. The current study utilize data collected between July and September 2019, approximately one year after the clinicians received training in screening and the treatment models. Participants did not receive any compensation for participating.

### Participants

In total 774 clinicians were invited to participate. The response rate was 49.5%, resulting in a sample of 383 clinicians across 47 different child and adult clinics. The average number of participating clinicians per clinic was 8 (SD = 3.35; range = 2–15). Overall, 71.8% were female and the average age was 43.0 (SD = 10.9; range = 25–68). About half of the sample had an educational background in psychology (49.1%), followed by medicine (15.8%), social work (13.4%), and nursing (8.0%). The rest had other educational backgrounds (13.7%). Average number of years in the current profession was 11.75 (SD = 9.57; range = 0–41), whereas average number of years at current workplace was 5.69 (SD = 6.67; range = 0–33). In total, 41.8% of the final sample reported having received training in evidence-based treatment for PTSD (TF-CBT, EMDR, or CT-PTSD) as part of the ongoing national implementation project.

### Measures

Three measures were used for the purpose of this study; the Implementation Climate Scale (ICS)[9], the Implementation Climate Measure (ICM)[3], and the Measure of Innovation-Specific Implementation Intentions (MISII) [22]. All three instruments were translated into Norwegian. An already translated version of the ICS was used [16], while the ICM and the MISII were translated with permission by the scale developers.

All three measures were adapted to the EBPs being implemented, (i.e. EBPs for PTSD). Each measure was introduced with a sentence defining what the EBPs for PTSD are; in child clinics this was defined as “EBPs for PTSD = using the screening instruments and TF- CBT” and in adult clinics “EBPs for PTSD = using the screening instruments, EMDR and CT- PTSD”.

#### Translation procedure for the ICM

A forward translation from English to Norwegian was conducted by the 3rd and 4th author and a member of the implementation team. All translators and back- translators have Norwegian as primary language and are fluent in English. The back- translation was conducted by an independent person outside of the research team. The back-translation was then compared to the original version and discussed with the scale developers. No major differences between versions appeared, with only small adjustments to the final Norwegian version. More details about the translation procedure can be found in Additional file 2.

#### Translation procedure for the MISII

A forward translation from English to Norwegian was conducted by the 3rd and 4th author. The back- translation was conducted by a master student in psychology whose primary language is Norwegian and who is fluent in English. The back-translation was then compared to the original version and discussed with the scale developers. No major differences between versions appeared and the first translation was accepted as the final version.

*Implementation climate scale* (ICS) [9] consists of 18 items divided equally across six dimensions 1) focus on EBP, 2) educational support for EBP, 3) recognition for EBP, 4) reward for EBP, 5) selection for EBP and 6) selection for openness). All items were scored on a 5-point scale ranging from 0 (“not at all”) to 4 (“to a very great extent”). All 18 items were worded to target EBPs for PTSD (*item example: “The use of evidence-based practice for PTSD is prioritized in this service”*). The Norwegian version showed good reliability with a Cronbach’s alpha of 0.87 and acceptable construct validity [16].

*Implementation climate measure* (ICM) [3] consists of six items, two items per sub dimension (expectation, support and reward), and were scored on a scale from 0 (“not at all”) to 4 (“to a very great extent”). The ICM was originally developed and validated in the USA in two organizational contexts. The study sought to investigate whether implementation climate could be measured as a global construct, using individual or group-referenced items, and whether the construct should be assessed at the individual or organizational level. The authors reported results supporting that the implementation climate is a global construct and that it should be assessed at the organizational level. However, they reported mixed results considering the use of individual or group referenced items. This depended on the context that the measure was used in. Their study showed acceptable internal consistency reliability and interrater reliability [3]. We used the individually referenced items in the Norwegian version of the ICM, and the item wording was adapted to the EBPs being implemented (*item example: “I get the support I need to use evidence- based practice for PTSD in treatment of my patients”).*

*Measure of Innovation-Specific Implementation Intentions* (MISII) [22] consists of three items with one item covering each of the three aspects of intentions: plans, desire and scope. Items were scored on a 5-point scale ranging from 0 (“not at all”) to 4 (“to a very great extent”). The scale shows good internal consistency with a Cronbach’s alpha of 0.90 and good person separation (PSI = 0.872) [22]. The authors suggest further examination of the psychometric properties of the scale such as criterion- related, discriminant, divergent and convergent validity, as well as sensitivity of responsiveness and using other innovations or EBPs [22]. The Norwegian version consisted of three items for MISII screening (*item example: “I plan to use [screening instrument] with my patients”*) and three items for MISII Treatment (*item example:” I plan to use TF-CBT / EMDR or CT-PTSD with my patients”*).

### Statistical Analyses

Confirmatory factor analysis of the ICS was performed with MPlus 8 [28] accounting for the nested data structure (TYPE = COMPLEX) and using Weighted Least Square Mean and Variance Adjusted estimation (WLSMV) appropriate for ordered-categorical indicators. Several fit indices were used to determine model fit: comparative fit index (CFI), Tucker-Lewis index (TLI), root mean square error of approximation (RMSEA), and standardized root mean square residual (SRMR). CFI and TLI values above 0.95 and RMSEA and SRMR values below 0.08 indicate acceptable model fit [29, 30]. A script of the analyses in MPlus is provided in the Additional file 1. All other statistical analyses were performed in IBM SPSS Statistics 26. We used Cronbach’s alpha to assess internal consistency reliabilities for the total scale and subscales, where values of 0.70 and above indicate that the internal consistency is satisfactory [29]. To evaluate criterion related validity, we performed several bivariate correlation analyses between the ICS (total scale and subscales) and the ICM and MISII scales Since the ICS presupposes that implementation climate is a group-level construct, correlation coefficients on both individual and unit level are reported.

## Results

### Confirmatory factor analysis

**Fig. 1:**
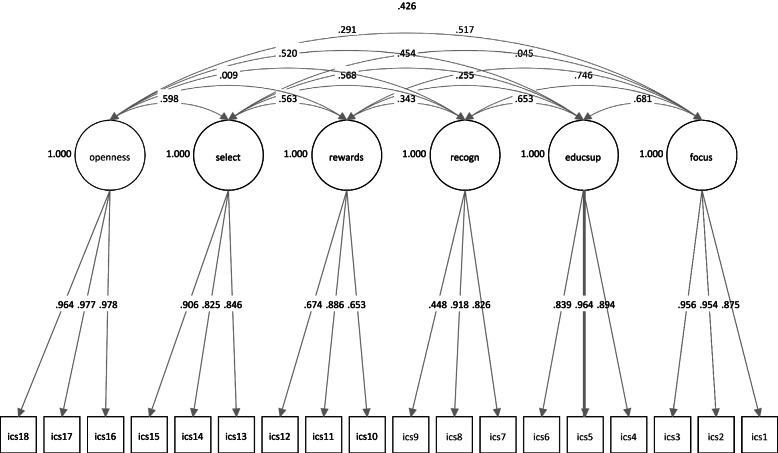
Confirmatory factor analysis model of the ICS

The confirmatory factor analysis tested a six-factor model with correlated latent factors. The items were forced to load onto their hypothesized factors. Overall, the model demonstrated good fit, although the RMSEA value was a bit high (χ^2^(120) = 444.467, p < 0.001; CFI = 0.985; TLI = 0.981; RMSEA = 0.084, 90% CI [0.076-0.092]; SRMR = 0.074). Standardized and unstandardized factor loadings for each indicator are provided in Table 1. Standardized factor loadings ranged from 0.448–0.978, with a mean of 0.855, all significant at p < 0.001 (Fig. 1). For item 9 (“*Employees that use evidence-based practice for PTSD have increased possibility for promotion in this service*”), the standardized factor loading (0.448) and R- square value (0.201; p < 0.001) were lower compared to the other items. This indicates that only a small share of the variance in this item is explained by the latent factor recognition. This, in conjunction with a high RMSEA value for the model, warranted further inspection of modification indices provided by Mplus. These suggested that allowing item 9 to load on the latent factor reward, in addition to the latent factor recognition, would improve the model significantly. When we ran the analysis again with this modification, model fit improved and RMSEA lowered to an acceptable level (χ^2^(119) = 342.897, p < 0.001; CFI = 0.989; TLI = 0.986; RMSEA = 0.070, 90% CI [0.061-0.079]; SRMR = 0.061). The R-square value for item 9 increased to 0.330 (p < 0.001). The other modification that would have led to significant improvement in model fit, allowing item 12 (“*This team/agency provides the ability to accumulate compensated time for the use of evidence-based practices”*) to load on the latent factor Selection for EBP, does not make sense theoretically, nor intuitively, and was therefore not explored further.

**Table 1:** Standardized and Unstandardized CFA Factor Loadings

ICS Factor items	Standardized Factor Loadings	Unstandardized Factor Loadings
**Focus on EBP for PTSD**		
1. Main goal is to use EBP effectively	0.875	1.000
2. Think implementation is important	0.954	1.090
3. Using EBP is a top priority	0.956	1.092
**Educational Support for EBP for PTSD**		
4. Conferences, workshops, or seminars	0.894	1.000
5. EBP trainings or in-services	0.964	1.078
6. Training materials, journals, etc	0.839	0.938
**Recognition for EBP for PTSD**		
7. Seen as clinical expert	0.826	1.000
8. Held in high esteem	0.918	1.111
9. More likely to be promoted	0.448	0.542
**Rewards for EBP for PTSD**		
10. Financial incentives for use of EBP	0.653	1.000
11. More likely to get a bonus/raise	0.886	1.356
12. Accumulate compensated time	0.674	1.031
**Selection for EBP for PTSD**		
13. Previously used EBP	0.846	1.000
14. Formal education supporting EBP	0.825	0.975
15. Value EBP	0.906	1.071
**Selection for Openness**		
16. Adaptable	0.978	1.000
17. Flexible	0.977	0.998
18. Open to new interventions	0.964	0.985

*N* = 383; 47 clusters

All loadings significant at *p*  <  0.001

### Summary statistics and correlations

Means, standard deviations and scale reliabilities for the ICS total scale, subscales, and scale items are provided in Table 2. The internal consistency of the total scale was 0.90 and ranged from 0.70 to 0.97 for the subscales, except for the Reward subscale which was considerably lower at 0.54. Correlations between the total scale and subscales at both individual-level and group-level are provided in Table 3. Results show moderate to strong correlations, except for the Reward subscale. However, the correlation between the Reward subscale and the total scale is moderate and significant at both levels (0.42-0.49; p < 0.01). It is also worth noting differences in mean values between items within the Recognition subscale and the Reward subscale. Within the Recognition subscale, item 9 (more likely to be promoted) has considerably lower mean than the two other items, whereas the opposite is the case for the Reward subscale where item 12 (accumulated compensated time), had considerably higher mean than the two other items.

**Table 2:** Summary statistics for the ICS total scale, subscales, and scale items

ICS total and subscales	Mean	SD	*a*
**Implementation Climate Total Scale**	**1.94**	**0.60**	**.90**
***Implementation Climate Subscales***			
**Focus on EBP for PTSD**	**2.61**	**0.88**	**.91**
1. Main goal is to use EBP effectively	2.60	1.00	
2. Think implementation is important	2.68	0.92	
3. Using EBP is a top priority	2.55	0.94	
**Educational Support for EBP for PTSD**	**2.00**	**1.00**	**.90**
4. Conferences, workshops, or seminars	2.02	1.07	
5. EBP trainings or in-services	2.04	1.14	
6. Training materials, journals, etc	1.94	1.06	
**Recognition for EBP for PTSD**	**1.99**	**0.81**	**.70**
7. Seen as clinical expert	2.29	1.00	
8. Held in high esteem	2.66	0.95	
9. More likely to be promoted	1.02	1.14	
**Reward for EBP for PTSD**	**0.70**	**0.68**	**.54**
10. Financial incentives for use of EBP	0.5	0.89	
11. More likely to get a bonus/raise	0.27	0.64	
12. Accumulate compensated time	1.32	1.22	
**Selection for EBP for PTSD**	**1.65**	**0.90**	**.87**
13. Previously used EBP	1.45	0.97	
14. Formal education supporting EBP	1.65	1.05	
15. Value EBP	1.86	1.01	
**Selection for Openness**	**2.69**	**0.90**	**.97**
16. Adaptable	2.68	0.96	
17. Flexible	2.71	0.91	
18. Open to new interventions	2.68	0.91	

*N* = 383

**Table 3:** Individual-level and group-level ICS total scale and subscale correlation matrix

	**1**	**2**	**3**	**4**	**5**	**6**	**7**
1. Implementation Climate Total Scale	–-	.88^a^	.80^a^	.88^a^	.49^a^	.87^a^	.66^a^
2. Focus on EBP for PTSD	.77^a^	–-	.70^a^	.74^a^	.25	.74^a^	.50^a^
3. Educational support for EBP for PTSD	.74^a^	.60^a^	–-	.70^a^	.34^b^	.54^a^	.30^b^
4. Recognition for EBP for PTSD	.78^a^	.58^a^	.53a	–-	.46^a^	.65^a^	.46^a^
5. Rewards for EBP for PTSD	.42^a^	.07	.19^a^	.25^a^	–-	.39^a^	.08
6. Selection for EBP for PTSD	.77^a^	.46^a^	.40^a^	.47^a^	.37^a^	–-	.67^a^
7. Selection for openness	.63^a^	.39^a^	.25^a^	.39^a^	.03	.49^a^	–-

Individual-level correlations (*N* = 383) are below the diagonal, and group level correlations (*N* = 47) are above the diagonal. ^a^*p* < 0.01, ^b^*p* < 0.05

### Criterion- related validity

Correlations between the ICS, the ICM, and the MISII scales are provided in Tables 4 and 5. The correlations between the overall scales of the ICS and ICM were 0.65 at the individual level and 0.79 at the unit level, both statistically significant. Correlations between the subscales, and between total scales and subscales, are also statistically significant and generally higher at the unit level than the individual level. At the individual level, correlations between the ICS total scale and the MISII Screening and Treatment scales are 0.18 and 0.15, respectively, with the latter bordering statistical significance with a p-value of 0.055. At the unit level, the correlations are 0.19 for MISII Screening and near zero for MISII Treatment, both statistically insignificant. Generally, all correlations between the ICS (total scale and subscales) and the MISII scales are in the lower range, with the largest correlation between Selection for Openness and MISII Screening (0.33) at the unit level.

**Table 4:** Individual-level correlations for criterion validity of the ICS

	ICS Total Scale	Focus on EBP	Educational support for EBP	Recognition for EBP	Rewards for EBP	Selection for EBP	Selection for openness
ICM Total Scale	.65^a^	.58^a^	.53^a^	.45^a^	.19^a^	.49^a^	.41^a^
ICM Expected	.47^a^	.46^a^	.35^a^	.28^a^	.17^a^	.38^a^	.29^a^
ICM Supported	.64^a^	.57^a^	.56^a^	.46^a^	.15^a^	.41^a^	.43^a^
ICM Rewarded	.61^a^	.50^a^	.50^a^	.45^a^	.18^a^	.49^a^	.37^a^
MISII Screening	.18^a^	.17^a^	.11^b^	.04	-.00	.14^a^	.25^a^
MISII Treatment	.15	.20^b^	.11	.06	-.05	.11	.17^b^

*N* = 160 for correlations with MISII Treatment. *N* = 383 for correlations with all other scales

^a^*p* < 0.01, ^b^*p* < 0.05

**Table 5:** Group-level correlations for criterion validity of the ICS

	ICS Total Scale	Focus on EBP	Educational support for EBP	Recognition for EBP	Rewards for EBP	Selection for EBP	Selection for openness
ICM Total Scale	.79^a^	.75^a^	.69^a^	.67^a^	.36^b^	.57^a^	.52^a^
ICM Expected	.66^a^	.71^a^	.54^a^	.49^a^	.40^a^	.47^a^	.41^a^
ICM Supported	.79^a^	.71^a^	.73^a^	.68^a^	.28	.56^a^	.56^a^
ICM Rewarded	.74^a^	.69^a^	.66^a^	.68^a^	.32^b^	.55^a^	.47^a^
MISII Screening	.19	.12	.13	.18	.03	.04	.33^b^
MISII Treatment	.02	.09	-.19	.10	.25	-.05	-.03

*N* = 45 for correlations with MISII Treatment. *N* = 47 for correlations with all other scales

^a^*p* < 0.01, ^b^*p* < 0.05

## Discussion

The goal of this study was to assess the psychometric properties for the Norwegian version of the ICS in clinical mental health care settings. We sought to investigate the six-factor structure of the ICS and internal consistency, as well as to evaluate criterion validity of the scale. Results showed acceptable fit for the six-factor structure of the ICS and strong internal consistency reliability for the total scale thus providing support for hypothesis 1 and 2. Furthermore, the pattern of correlations with the ICM demonstrated the concurrent validity of the ICS, with stronger correlations at unit level compared to individual level, supporting hypothesis 3. Finally, although criterion- related validity analysis revealed mostly positive correlations with the MISII, these were not statistically significant, thus we did not find support for our last hypothesis.

Overall, the results support the psychometric properties of the total ICS scale, although findings for the Reward subscale were not as strong as for the other subscales. This finding is consistent with previous studies [13–16]. As argued in these studies, one likely explanation for this is limited resources and opportunities for providing financial rewards. In Norway, financial rewards or incentives are rare, if not completely absent, in the public mental healthcare system. Thus, the relevance of the items in the Reward subscale likely had an impact on the reliability of this subscale in our study. The pattern of responses nevertheless indicates that one of the three items of the Reward subscale, item 12 (accumulate compensated time), seemed more relevant for respondents (larger mean compared to other two items). Furthermore, compensation in accumulated time is an actual possibility for clinicians, but not necessarily specifically connected to EBP. This is a general rule in Norwegian mental health clinics, originally introduced as a work environmental initiative.

In contrast to the Reward subscale, the Recognition subscale performed considerably better, even though the alpha value was lower (α = 0.70) compared to previous validation studies (α = 0.77-0.88) [9, 15]. In this case, pattern of responses on item 9 (more likely to be promoted) indicate that this item might be less relevant in a Norwegian context. Promotion would normally involve a financial bonus or raise, thus making this possibility unlikely solely based on EBP knowledge and performance. It may therefore be perceived as a financial reward. Clearly, there seems to be a conceptual connection between reward and recognition [9]. Combining the results on these two subscales can potentially have implication for adaptation to improve the fit of the ICS in a Norwegian mental health setting. Ehrhart, Torres [15] note that removing the Reward subscale would improve the ICS performance for research purposes, but that focus on reward might still be important for practical implementation efforts, thus arguing for applied purpose for inclusion of the subscale. Since the results of the Reward subscale are lower in our study compared to validation studies in the US, this could indicate some cultural and/or organizational differences, and the question is whether adaptation of the scale will have an impact not only for research purposes, but also for practical implementation efforts. One possibility is to remove the Reward subscale while rephrasing the Recognition subscale, especially item 9, towards non-financial recognition. Another possibility could be to keep the Reward subscale but rephrasing financial rewards towards rewards that are suitable and realistic within organizational resources. This should be further investigated in future studies. This adaptation could make the ICS more organization/sector specific, thus more useful for practical implementation purposes, but possibly less useful across organization/sectors. This implicates that ICS should be adapted when used in different setting, as also suggested by Lyon, Cook [13].

In investigation of concurrent validity of the Norwegian ICS in comparison to ICM, we found stronger correlations at the group level than the individual level. This might be due to differences in specific versus general items, as originally pointed out by Ehrhart, Aarons [9]. ICS items focus on specific policies, practices and procedures in the organization, i.e. “what I perceive actually happens in the organization considering implementation climate” versus general perceptions of implementation climate, i.e. “how I perceive the organization’s climate for implementation”. As discussed above, this might further make the ICS more relevant as an organization specific tool and hence useful for specific feedback on what leaders do and communicate. Furthermore, while the ICS operationalize reward as financial incentive/raise/time compensation, the ICM operationalizes reward as recognition/appreciation. This also brings us back to reward and recognition being conceptually connected constructs.

Our last hypothesis, expecting positive correlations between implementation climate and the clinicians’ intentions to use EBPs was not supported. Even though there were mostly positive correlations between implementation climate as measured by the ICS and clinicians’ intentions to use the EBPs, most correlations were not statistically significant and some even negative at both individual and group levels. At both levels this might partly be due to the fact that all clinicians responded to the survey, not only those that received training in the EBPs. Thus, questions about intention to use EBPs for PTSD might have been less relevant for clinicians that did not receive training in EBPs for PTSD, even if all clinicians received training in trauma and PTSD screening. Each item in the MISII refer to the specific screening instrument and specific evidence- based method. The ICS items refer to “EBPs for PTSD” and even though these are defined at the start of the survey, not specifying each item might have had an impact on how “EBP for PTSD” was understood and interpreted. Thus, it might be relevant to specify each item to the specific screening instrument and method. At the unit level, these correlations might also be due to low number of observations. The largest correlation between the ICS subscales and the MISII Screening criterion variable is the Selection for Openness subscale. This subscale is the most general, as the items do not ask for EBPs for PTSD and might thus be more relevant also for clinicians that did not receive training in EBPs.

Intentions are found to be a valid proxy measure for behavior when implementing new interventions [31].How strong this intention is depends on attitudes towards the behavior, subjective norms and perceived behavior control [19]. Through our contact and practical work with participating clinics in this study, clinicians reported multiple factors that were influencing their use of the EBPs, such as having enough time for all their patients, having too many patients to treat, availability of materials such as light machines for EMDR practice, insecurity about how patients would react to the screening or the treatment, etc. These factors could also have contributed to how clinicians perceived behavior control, thus also influencing intentions to use EBPs. It is surprising that our finding shows week association between implementation climate and intentions to use, and future research should examine this link further, e.g. in a predictive validity study. Future research should also measure clinicians’ actual behavior and the association to implementation climate as measured by ICS. This can contribute to more knowledge for practical purposes of organizational preparations for implementation efforts.

The need for high-quality implementation outcome instruments is identified as a critical gap in the implementation literature [32]. By investigating implementation climate in a Norwegian mental healthcare setting using the ICS, this study also contributes to generalizability of the scale across cultures. Furthermore, since the ICS version in our study focused on specific EBP, this study provide evidence for the psychometric properties of the ICS assessing climate for specific EBP. Yet some limitations of our study should be noted. First, even though our sample size was acceptable at the individual level, it was small at the group level. Second, our sample consisted of all clinicians receiving training in EBP assessment tools, and some clinicians receiving training in EBP treatment methods. It is unknown how *not* receiving training in EBP treatment methods might have influenced clinicians’ responses on implementation climate for EBPs, i.e. how relevant they might have perceived questions about specific rewards and recognition for using EBPs. Furthermore, even though the goal was that all clinicians should receive training in trauma and posttraumatic stress screening and assessment, including training of new employees (a responsibility of the clinic leader), there might have been employees that did not. Therefore, it is unknown how this might have influenced their responses on perceptions of implementation climate. Third, in our study, the item wording in the ICM items were at the individual level while the item wording of the ICS was at group/organizational level. Wording of items could influence both variability in a construct and relationship between a construct and outcome [33, 34]. As difference in item wording might contribute to assessing related, but nevertheless different constructs, it is recommended to use group referenced items, rather than individual referenced items [1].

In organizations that implement multiple EBPs, as in our study, further research should investigate responses of clinicians that practice EBP treatment versus those who do not, and how this might affect their perceptions of implementation climate of a specific EBP in the organization. Also, adapting the modifications to ICS suggested above would be useful to test in order to see how this would have relevance for the practice field, in addition to the research field. Future studies should also seek to measure clinicians’ actual behavior in association to implementation climate.

## Conclusion

Findings from this study suggest that the Norwegian version of the ICS can be useful for mental health care organizations in evaluating climate for implementing EBPs. It can offer a way to identify which aspects of the climate specifically to attend to when preparing and implementing EBPs. Since the Reward scale performed poorly in the Norwegian mental healthcare setting, adaptations of the Norwegian version of the scale are recommended and should be tested.

## Supplementary Information

**Additional file 1:** Confirmatory factor analysis of the ICS in MPlus

**Additional file 2:** The translation procedure of the Implementation Climate Measure (ICM).

## Data Availability

Data is stored at Norwegian Centre for Research Data. Data cannot be shared, as the consent for publication of the dataset has not been obtained and because of sensitivity of information being given.

## Abbreviations

EBPs: Evidence-based practices
ICS: Implementation Climate Scale
ICM: Implementation Climate Measure
MISII: Measure of Innovation-Specific Implementation Intentions
CFA: Confirmatory factor analysis
PTSD, PTSD: Posttraumatic stress disorder
TF-CBT: Trauma-focused cognitive behavioral therapy
CT-PTSD: Cognitive therapy for PTSD
EMDR: Eye movement and desensitization reprocessing
