# Supplementary material for: Measuring implementation climate: psychometric properties of the Implementation Climate Scale (ICS) in Norwegian mental health care services
Source: BMC Health Serv Res. 2022 Jan 4;22:23. doi: 10.1186/s12913-021-07441-w (PMC8725247; doi:10.1186/s12913-021-07441-w)
Supplement: Supplementary file 1 — Additional file 1: Confirmatory factor analysis of the ICS in MPlus [file 12913_2021_7441_MOESM1_ESM.docx]

## Additional file 1: Confirmatory factor analysis of the ICS in MPlus

TITLE: CFA ICS

DATA: FILE IS datamplus.dat;

VARIABLE:

NAMES ARE klinikk2

ICS1

ICS2

ICS3

ICS4

ICS5

ICS6

ICS7

ICS8

ICS9

ICS10

ICS11

ICS12

ICS13

ICS14

ICS15

ICS16

ICS17

ICS18;

CLUSTER=klinikk2;

USEVARIABLES ARE

ICS1

ICS2

ICS3

ICS4

ICS5

ICS6

ICS7

ICS8

ICS9

ICS10

ICS11

ICS12

ICS13

ICS14

ICS15

ICS16

ICS17

ICS18;

CATEGORICAL ARE

ICS1

ICS2

ICS3

ICS4

ICS5

ICS6

ICS7

ICS8

ICS9

ICS10

ICS11

ICS12

ICS13

ICS14

ICS15

ICS16

ICS17

ICS18;

MODEL:

focus by ICS1 ICS2 ICS3;

educsup by ICS4 ICS5 ICS6;

recogn by ICS7 ICS8 ICS9;

rewards by ICS10 ICS11 ICS12;

select by ICS13 ICS14 ICS15;

openness by ICS16 ICS17 ICS18;

ANALYSIS: ESTIMATOR IS WLSMV;

TYPE=COMPLEX;

OUTPUT: modindices(10) standardized;
