# Supplementary material for: Measuring implementation climate: psychometric properties of the Implementation Climate Scale (ICS) in Norwegian mental health care services
Source: BMC Health Serv Res. 2022 Jan 4;22:23. doi: 10.1186/s12913-021-07441-w (PMC8725247; doi:10.1186/s12913-021-07441-w)

# The translation procedure of the Implementation Climate Measure

Jacobs, S.R., Weiner, B.J. & Bunger, A.C. Context matters: measuring implementation climate among individuals and groups. *Implementation Sci* **9,**46 (2014). <https://doi.org/10.1186/1748-5908-9-46>

## Contents

### The description of the translating procedure.

### The First translation: from English to Norwegian.

### The Back- translation: from Norwegian to English.

### The Final version.

### Correspondence with the scale developers.

## Description of the translating procedure

Translation from English to Norwegian:

Mathilde Endsjø, Karina Egeland, and Ane-Marthe Solheim Skar at the Norwegian Centre for Violence and Traumatic Stress Studies (NKVTS) February 2019.

Back translation:

Kristine Dragland, Norway, February 2019

Scale developer reviewing the back translation:

Sara Jacobs, February 2019

Steps undertaken in the translation process:

1. Three project team members conducted translation from English to Norwegian. All have Norwegian as their mother tongue and are fluent in English. Two of the translators are researchers within the field of implementation science.
2. An independent person outside of the organization conducted the back translation. This person also has Norwegian as primary language and is fluent in English. Back translation was done without any knowledge or viewing of the original scale.
3. The back translation was e-mailed to the scale developer for review.
4. No discrepancies were found and the scale developer approved the back translation.
5. The first translation was used as the final version.

## First translation: from English to Norwegian

1. Det er forventet at jeg skal bruke [treatment] med et visst antall pasienter
2. Jeg er forventet å hjelpe min arbeidsplass å oppnå sine mål i implementeringen av [treatment]
3. Jeg får den støtten jeg trenger for å identifisere passende pasienter for [treatment]
4. Jeg får den støtten jeg trenger til å bruke [treatment] i behandlingen av mine pasienter
5. Jeg mottar anerkjennelse når jeg bruker [treatment] i behandlingen av mine pasienter
6. Jeg blir verdsatt når jeg bruker [treatment] i behandlingen av mine pasienter

0 Ikke i det hele tatt

1 Til en viss grad

2 I moderat grad

3 I stor grad

4 I veldig stor grad

## Back translation: from Norwegian to English

1. It is expected that I should use a [treatment] with a certain number of patients.
2. I am expected to help my workplace to achieve its goals in the implementation [treatment]
3. I receive the support I need in order to identify suitable patients for [treatment]
4. I receive the support I need in order to use [treatment] in the treatment of my patients.
5. I receive recognition when I use [treatment] in the treatment of my patients.
6. I receive appreciation when I use [treatment] in the treatment of my patients.

0 Not at all

1 To a certain degree

2 To a moderate degree

3 To a large degree

4 To a very large degree

## The Final version

1. Det er forventet at jeg skal bruke [treatment] med et visst antall pasienter.
2. Jeg er forventet å hjelpe min arbeidsplass å oppnå sine mål i implementeringen av [treatment].
3. Jeg får den støtten jeg trenger for å identifisere passende pasienter for [treatment].
4. Jeg får den støtten jeg trenger til å bruke [treatment] i behandlingen av mine pasienter.
5. Jeg mottar anerkjennelse når jeg bruker [treatment] i behandlingen av mine pasienter.
6. Jeg blir verdsatt når jeg bruker [treatment] i behandlingen av mine pasienter.

0 Ikke i det hele tatt

1 Til en viss grad

2 I moderat grad

3 I stor grad

4 I veldig stor grad

## Correspondence with the scale developers.


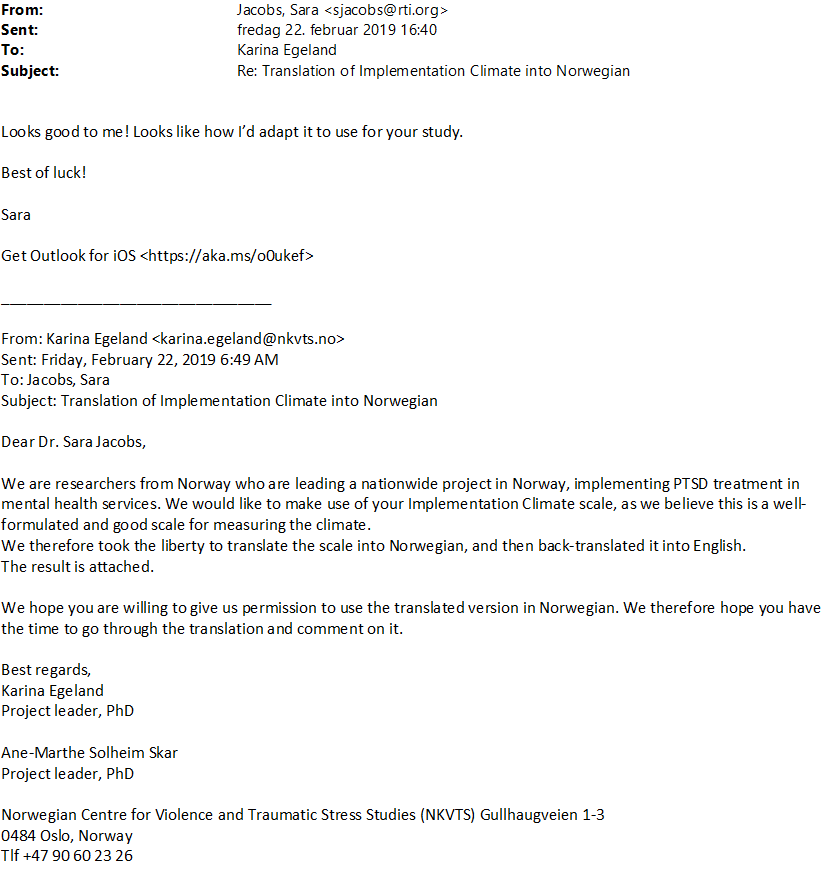

Supplement: Supplementary file 2 — Additional file 2: The translation procedure of the Implementation Climate Measure (ICM). [file 12913_2021_7441_MOESM2_ESM.docx]
